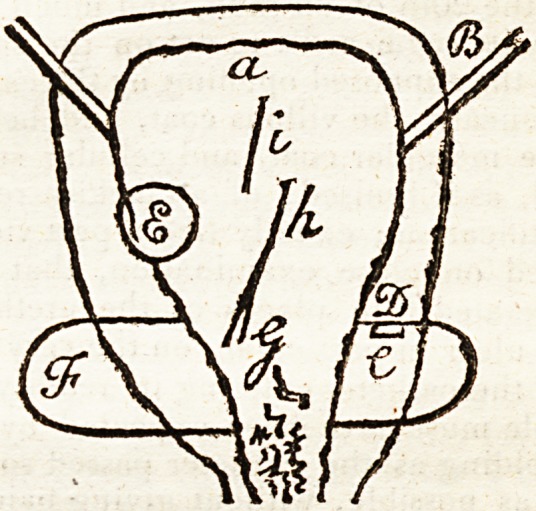# Dr. Bellamy's Case of Diseased Bladder

**Published:** 1807-12

**Authors:** 


### *Dr.* BELLAMY'S *Case of Diseased Bladder.*

[ Continued from Vol. xvi. pp. 141—149. ]

YOU will please to observe, that ever since his confinement to bed, he fell off in strength very fast; indeed, much quicker than could be expected for even a weakly man; therefore, extraordinary for so stout a person, after only two days in bed. I saw, as he lay, and even the day before he took to bed, when last he came to my cabin, a droll unaccountable state, which I rather referred to drink; a kind of half muttering, with weakness, looking pale, and a degree of deadness of the eye. I say this state of apparent debility, possible as it is to be connected with subsequent disease, and the possibility of new appearances, if the mortification be from influence of typhus, yet was more reasonably referred to drink, and laying in bed; for why did not that prostration and disease advance? On the contrary, he decidedly got better, after the abscess broke; acquired a little appetite, and had no sign unequivocal of fever, nor the least inconsistence, thirst, restlessness, heat, or one affection of the system worth notice; but, for so strong a man, is paler, weaker, softer, and more reduced, than is usually to be accounted for by an abscess. All this was referred to being in bed, previous abstiabstinence, and having now only a spare appetite; obliged to live low, while abscess forming; and since I have prompted him to take more, is rather indifferent; though all the house materials are regular. Decidedly no fever, pulse moderate, neither full, hard, quick, nor strong; nor has he had the least sign of inflammatory diathesis: on the other hand, he has not had that prostration and feebleness of pulse, to shew apprehension of typhus. I wished to see pulse firmer and fuller; therefore, to promote strength, and digestion of wound, gave the bark, encouraged him to eat, &c.; yet, with all this, he has continued pale, and with rather a deadly eye, a white tongue; and always, when he got up for a few minutes to have his bed made, or being in a particular posture to be dressed, was quite giddy and half faint; this every day last week, at which he always expressed his surprize; explained to him, by me, to long laying in bed; yet I have had others lay twice as long, and of weaker constitutions, and not feel any thing like such degree of vertigo and faintness; but as all this might be placed to the simple cause mentioned, it gave no uneasiness; particularly as he slept well, took courage, and thought himself better; and I thought so too (I speak of the abscess) till the water found its way through the perinæum, &c. As he ate tolerably, and the wound was so healthy, he was reading greater part of the day, and talking of getting up soon; which I intended he should, so soon as the poultice was left off, which I expected in a few more days.

Now, we know that typhus is a very insidious, and sometimes slow approaching disease; the patient unwell, and can hardly tell how long, probably a week; still is so unwell as to complain, which he has not done, of pains, oppression, restlessness, &c. Let us, however, remember, that he has been laying near two patients decidedly diseased, of no inconsiderable typhoid infection; recollect, also, the fetid breath, the action of mercury was so quick and lasting upon him: at the same time, let us bear in mind the preventive power of bark, which he has taken three or four days; this would check, on one hand, what he might lose on the other; in short, how are we possibly to account for the serious and sudden change of appearances, but by reference to the cause of low fever, acting on locally diseased parts? We know that, under such fever in particular, old wounds break out; blisters often ulcerate, and recent wounds will partake of the general disposition for gangrene; yet I cannot bring myself to think, that that such sudden change, both general and local, could take place from fever, without having previously distinctly seen that fever existed.

Now, on the other hand, is it not impossible to explain mortification of a part, or parts, and then refer all constitutional symptoms of debility to the influence produced on the system by gangrene of some important part? for it must be some important part, to produce such symptoms all at once; for do we not see a whole limb run into gangrene, and yet the patient only loses strength gradually; but here we have all the prostration, and every thing but hiccup and delirium, attendant on a sudden change, induced by mortification of intestine, or stomach, or bladder, or other great organ. But can we have it in a patient almost at death's door, who was as well yesterday evening before eight o'clock, as he has been this week past? I say now we have putrid symptoms, and gangrene of a viscus, probably without previous inflammation, or, at least, without the usual signs of such inflammation to disorganize it.

I have shewn that he has never had the least sign of inflammation, either general or local, since the formation and opening of the abscess, and even that abscess formed full of matter, without a rigor, or any usual precursor of inflammation, fever, &c. How then is this organ, the bladder or other, so disorganized?—On the whole, I must conclude that it is either typhus quickly brought to an acme, after having been latent some time, or else it is distinct mortification, and I fear of the bladder. But the mystery here is greater than admission of typhus, because where has been the previous inflammation?

He continued as well as usual all yesterday, eat and drank in common, made water occasionally in a small stream; but he said not so small, with more ease, and quicker done; though, when the desire came, he could not retain it, but only a sense of a drop or two came through perinæum. This ease he referred, in part, to use of the bougie in the morning; which only left a little soreness, as is usual. The wound looked exceedingly well in the evening, a little acrid pus, somewhat filled up, and contracted.

I proceeded to act on the principles yesterday spoken of, but could not succeed with the stillette of the elastic gum catheter, which was not sufficiently flexible to conform to the necessary curvature through the strictured parts, nor to be insinuated through the obstructions: it was was well oiled, and I employed a little force, though far from severe, and the patient scarce minding me. I gave it a second trial, after bending it like a catheter, but was equally unsuccessful; and although the patient did not particularly complain, I gave up its application, and proceeded to try the catheter, both as a sound to feel it in the perinæum, and as a bougie. At this time the bladder was empty, though he had not made water since 2 P. M. and had taken fluids. The catheter passed in with the usual degree of force; always a little pus, and a small quantity of blood, came out. I passed the handle over to the right groin with a degree of pressure, so as to lay it down a little; and it gave the sensation, as though it were *embraced* by the bladder. The patient spoke a little of pain, and the operation being longer than usual, he felt a little exhausted; but I cannot convince myself that any unusual force, or, at furthest, any force liable to do harm, was applied.

Could a small catheter do more harm than a bougie? Is there any particular evil in passing a bougie or a catheter, when the bladder is empty? Yes, you will say, because you may touch the coats; but is not the bladder divided in Lithotomy, and other necessary and accidental divisions of it, with impunity? Did I not only, yesterday, read of puncturing it with a trocar, when in a high state of inflammation and distension, and the trocar retained in it a whole week? Was there any particular evil in passing the stilette of the elastic gum catheter? It did not go further than the arch of the pubis, and no particular force or pain was given. Yet I allow, from the combined efforts, it was not unlikely to have inflammation follow; I even began to think I should have to evacuate him, if I followed up, as I intended to do, the principle of exciting a degree of inflammation; and as I hoped the consequent healing process, by retention of a bougie, or now by that of the catheter; but on the whole I preferred this view to an operation, and so confined the catheter pretty deeply inserted in the bladder, right in the centre of the pubis, by a tape. Dressed, poulticed, and gave the bark, and left him at seven, P. M. Saw him again at nine; found him just settling in bed, after being on its edge to make water. I had left word, should he want to make water, to withdraw the stilette. He was never left by one or other of my assistants, and the catheter was not to be drawn without my knowledge; for I had even an idea of keeping it in all night. He had a moderate desire to make water water for about five minutes; the assistant begged him to put it off till I came, but it became so pressing, he could not. The stillette was withdrawn; he sat on the edge of the bed; not a drop of water came through the catheter, but he absolutely passed the whole (a half pint) by the sides of that instrument, in a tolerable stream, but gave great pain and straining, yet not one drop went through the perinæum; however, with this exertion, he felt very faint, and on my arrival, just as he lay down, a violent rigor, equal to that of an intermittent, came on, lasted with violence half an hour, and moderately an equal length of time; but has never to this moment, ten A. M. 1 Feb. been followed by heat or reaction of any kind. I begged him not to be alarmed, and withdrew the catheter; it came out with the greatest ease; and all the time it had been in (about two hours) he had lain as easy as usual, felt no pain, but a sense of fulness of the instrument; expressed no desire to have it withdrawn, or any one symptom or uneasiness, till the desire to make water. A little blood came out on withdrawing the catheter; we suspected the reason of urine not passing through it, was coagulated blood, but there was not a bit of coagulum in the strainer.

Is it not wonderful, therefore, that the urine should flow by the sides of the instrument? Is it possible, with the little force applied, that the catheter went through the bladder? The force applied could not do this under common cases. Is it probable that the bladder was previously half dissolved, soft, and giving way by ulceration, &c.? Then, surely, he would have felt some pain, by the point of the catheter going into the abdomen; and urine would also, in part, have gone through: likewise, while inserted, the least touch seemed to bring it up; whereas, by going through, it would have passed on too freely. But I have stated facts. This rigor was then a mystery; how much more so now? Rigors usher in formation of matter when so violent, slighter rigors precede inflammation; and also even severe ones may precede fever of typhus kind, but never known to harbinger gangrene. If matter was to be the consequence, catheter had nothing to do with it, nor the fever of typhus, but as introductory to fever of irritation; though not common, and very uncommon to be so violent for inflammation; yet I dreaded that accident, and apprehended I had excited too much, fearing I should have to reduce a patient already weak.

For these few days past, and this evening in particular, when first dressed, he spoke of debility and giddiness; and and more so to night: put a plenty of clothes over him, gave opium gr. ij. applied a fresh poultice, &c. My mind was made up for excess of inflammation, which I expressed, and assistants also thought; a quarter of an hour after, he rejected the contents of the stomach, and with it the opium, no doubt; he felt faint, the pulse rather weak, being not yet completely over the cold fit: left word to keep stomach empty at present, but to repeat opium gr. ij. if restless, in pain, or distressed. About half past ten, he had a copious well formed stool, passed his urine, but not through perinæum: he got out to do it, as he could not manage it in bed; felt faintish, weak, and low; then slept a few hours, and apparently at ease, but no reaction, even of heat. About twelve he had a copious loose stool, and made water out of bed, and was more faint. Dressings were re-applied, and he appeared to fall asleep again; pulse about ninety, and tolerably good; no apprehension in the least entertained, or expressed to me by assistant, when I had a report at midnight, though I was very minute in my enquiries. He passed the middle-watch apparently at ease, and mostly asleep, no groans, or complaint; but when he awoke was faintish, and pulse very weak; it gradually sunk; and was reported to me at four A. M. to be very weak indeed, scarcely perceptible at times, yet no sense of absolute danger intimated to me; all this very strange! Gave up my fears of inflammation, but little dreamt of gangrene. He had neither stool nor urine all that watch; and assistant reported that, on the whole, he was all that time nearly as he found him; (observe there was a different assistant every four hours); that even pulse, at one A. M. was nearly as weak as when he came off watch, and not the least idea of danger was conveyed to his mind about him. He gave the opium gr. ij. at half-past one, because he was rather restless; but, lo! in the morning, about seven, first assistant represented, to my great surprize, the extreme prostration, particularly of the pulse; that it absolutely was not to be felt, but at times; and, above all, he confirmed this phenomenon, by state of the wound; the dressings had slipped off, and he saw the wound absolutely livid, and approaching to gangrene, though he did not then so express it; made no confession, but merely said, and, "Sir, the wound looks blackish, very nasty indeed."

I started up in a moment, and found as described before, decided, apparent, or rather well advanced gangrene; no pulse, skin rather cool, though as much as most persons persons natural heat, but not so hot as he ought to have been under common circumstances of least degree of inflammation, so contrary, indeed, to what I had expected, of a very high degree of it; face not sunk, the eye rather so; intellect clear as ever; a little mutter in the manner of speech, as if his mouth was dry; great thirst, tongue moist, but blackish.

This is a most inexplicable business, to have undoubted signs of general and local mortification, without preceding inflammation. If from fever, that has never been well marked till now, that it is very distinctly of the putrid kind, all at once; the local gangrene could never cause such symptoms so suddenly; and what could induce the local gangrene? If we look back to the case, nothing but the prostration of putrid fever. No, depend on it, the local state is the effect either of fever, or the mortification of some great viscus. Then the mystery is, has such mortification of viscus taken place, without preceding inflammation? Yet, on the whole, I take it for certain, that the local appearance is the effect either from fever disorganizing those parts in particular, because previously wounded.

Great and inexplicable are the difficulties. All I did with instruments would lead to inflammation, and that seemed indicated by the very severe rigor. But, no; it now appears that the rigor was the effect of sudden solution, and breaking down of the circulation and tone of the system, of which there was not much remaining, as seen by the general disposition ever since his illness, and by the abscess forming, without the fever of inflammation or rigor; but either as a deposit, or falling down of matter from elsewhere on a weak part;—begun to treat immediately as for gangrene and typhus, and considered that there was more than usual debility, a little mutter, &c. from the strong sedative force of opium, admitting he had retained three grains; for I believe it will be allowed, that there are habits on which opium will, at once, act sedatively, though generally first by stimulus, and consequent sedative affection, from over excitement, as from spirits, wine, &c.

This is a mere suggestion of the moment, but has nothing to do with the indications now to be pursued; for allowing all the general symptoms to be accidentally induced, we have eye-witness of local gangrene. Applied fermented warm cataplasms every two hours; camphor gr. iij. opium gr. ij. altern. 4tis horis; strong wine ℔j, infus infus. cinchon. ℔j. die.; in short, to take of bark as much as his stomach could bear, eighteen gr. camphor, and six of opium, in twenty-four hours. He began immediately, and continued this course tolerably well till afternoon, when he began to reject the bark, and soon after the wine and camphor; and uncertain if even the opium, or any thing of consequence, was retained; and he had every sign of exhaustion. In the evening, after the action of vomiting, he had three hiccups, apparently from the acidity of the wine, and emptiness of the stomach as to solids; but by what was retained, which might be three grains of opium, nine of camphor, vin. and cinchon. aa. ℥iv. not the least heat or re-action was produced, nor any alleviation whatever of prostration; on the contrary, he fell very gradually weaker, the pulse indistinctly to be felt like a thread for a few beats, at intervals, and chiefly when he had coughed. Observed he had complained of it, though seldom heard by me; he has said, "this nasty cough tears me." It is that peculiar cough which frequently attends putrid fever; a sort of convulsive act, for a few respirations, denoting anxiety and oppression of the chest, rather than a pathognomonic cough. It has now distinctly increased; he pants it out, as it were, but spits sometimes a thick whitish mucus, of a fetid smell. I considered, by the little I had seen and heard of it before, that he had a little attending cough of the prevailing catarrh, and most of the spitting to depend on the influence of mercury. There was scarce any alteration all the day or night, but a slow increase of the gangrene, with a proportionate spreading of the erysipelatious redness around, and some more small vesicles, even on the scrotum; no fœtor from the sore or body, but by night had lost a great deal of the almost natural degree of heat, the only satisfactory appearance hitherto remaining. Remember, as I said before, there was no hiccup as a formed symptom, nor delirium; the eyes a little more dead, the tongue moist, but collects a black sordes. He took some tea and a bit of bread for breakfast, and at dinner a small bit of animal food; in the evening, half a pint of sago.

I pronounced great danger from the first; scarce a probability of recovery; and knowing, that if not better by the morning, he would be worse, or dead: rather, however, thinking, that if he lived till the morrow, with mortification of the viscera, (concluding that the bladder alone was not sufficient to occasion such symptoms) that theretherefore death would quickly follow, or else he would recover, by being greatly relieved, and from the extent of the gangrene.

I dressed him at two in the morning of the 2d instant, thought the wound looked a little better, some little sign of redness, and absence of the general blackishness, and dryness on the whole; yet the surrounding redness is increased, no alteration of symptoms, and I wonder, being so very low, that he has existed so long: greater part of the time no pulse, and when to be felt, is at least 110; the loss of heat is now extended to cold clammy sweats, and great softness of the skin; no absolute delirium, the eyes mostly fast, little twitches; on the whole is declining, and there is no hope.

Now a new idea arises; still has not made a drop of urine since the night before, and yet has never expressed or felt the least desire; nor is there any fullness of the abdomen, or region of the bladder, but quite flat, soft, and flabby, and without sensibility to pressure; in short, there is a common absence of all pain, and it is remarkable how little he has had since the abscess broke, except when dressed or probed, and straining to make water; yet he has taken, since the period mentioned, full three quarts of fluid, though I have corrected his desire for drink, which was very earnest till noon yesterday. This is a very inexplicable circumstance in pathology; for if the bladder has lost all tone by gangrene, why does not the urine flow involuntarily; and if in it, surely must give pain and fullness; if not mortified, it only remains to suppose that it flows into some other cavity, that the parietes of the bladder are broken, and so as it enters the bladder, it falls into the cavity of the abdomen, or of the pelvis. In the former, it could not be without producing fullness and pain, unless, as previously supposed, the bowels are also gangrened. In the pelvis, I think it could not be in so great a quantity, without giving a sense of fullness, especially on the rectum, and some pain, unless those parts also are gangrened; and in either it must produce fullness. But no, all is slack and soft, as if quite empty. We may allow this, that the general contents of the abdomen are mortified; and as he is a large man, and bowels long since well cleared out by abstinence, &c. so water to that amount may be in the interstices of intestines, without producing a visible fullness. We may, to be sure, go further in our ideas, and suppose that there is no secretion of urine; that is to say, the emulgents, or kidneys, or urethers, or all are impervious; then it must be from gangrene also, else there would be great pain, sense of pressure, fever, &c. without you go back to a total annihilation of the emulgents before, and where they are given off, and so that all the fluids concerned are taken up into the circulation; no secretion of urine being made therefrom, part of the fluid so to be excerned may be in general circulation, the rest in the intestines; which are unable, by gangrene, to throw off their contents, having been so long without evacuation; when last, both were ample, and the stool very watery. When I speak of fluid to be drank, I mean that he has retained three quarts, having allowed for vomiting and waste. Taking all these circumstances together, it is plain there can be no water in the bladder; and it is highly probable that viscus is gangrened, and probably broken; which may have happened in three ways, by the breaking down of gangrene, or by ulceration and highly probable state long previous, and likely existing coeval with the disease of the urethra, now by really putrid diathesis giving way in those weak ulcerous parts, and afterwards fully mortified. Third way, is the possible rupture of it by the catheter. This is possible, but not very probable; for there was no unnatural force, nor, I believe, aukwardness employed; yet moderate force upon a part previously ulcerated, and hastening to gangrene, might effect it; for gangrenous solution must have been advancing some days insidiously, as seems confirmed by the general disposition to debility, head-ach, faintness, vertigo, and a peculiar cough; for it is absolutely impossible to impute the date of the cause of these sad effects to the period of passing the catheter; and if it could, then surely there must have been inflammation first.

It may be divided thus; that there has been all along a slow insidious approach of gangrene, from ulceration of the bladder, first; communicated since to the system in the form of typhus, very indistinct, though till at this time; and on the bowels also by proximity. Then also we may condemn, and say the catheter assisted the catastrophe; not by repeated irritation, but by helping to break through the most diseased parts; and thus also, and the only way in which I can account for it, the water not coming off through the catheter, but by its sides; because it might have gone through the bladder, the length of the strainer into the abdomen, and yet fit so nicely, that most of the urine should, at that time, flow by the urethra; and, in confirmation of this disorganization, remember the pressure of of necessity constantly to make it, especially at the time of the catheter being in; and I scarce doubt that at both times after, when he had stools, that most of the urine then flowed involuntarily, and for the last time; because its cavity since became obliterated. Also, at these two last times, a partial tone might exist to evacuate the water; and part might escape through, where the catheter had been. Dissection alone can decide.

To my great astonishment, he survived through the night of the 2nd of February, with the following circumstances:—From eight to twelve, almost a continual short cough, bringing up great quantities of thick, and rather blackish fetid mucus, with scarce any power to spit it out; and though a very slow, yet a certain progressive advance to death; the general fœtor and flabbiness of the flesh increasing, face sinking, eyes seldom open, no power of speech or deglutition; and when not coughing, a severe action of the stomach to eject great quantities of highly fetid, thin, black fluid; evidently not only the contents of the stomach from the great quantity of it, but also of the intestines; and I have now, fully to my mind, explained the absence of urine, tension, or fullness of abdomen, and lower region, on the principle of want of absorption; and that all the fluids received, have been kept in the small intestines and stomach, and become black by mixture of bile, the secretion of gastric juice, and that of the intestines, as well as of the first formed fœcal matter in the first intestines, and so much more putrid by the hasty strides; or, you would say, complete state of malignancy, and solution by gangrene.

The putrid exhalation has become so strong these last thirty hours, as greatly to affect all the attendants; and one man now fainted away, when breathing over him. Nothing could be got down; and, indeed, it would be only temerity to attempt it. The ulcer in perinæum is quite dry, and positively for a considerable extent taking in the anus, and quite dead and soft, like a part cadaverous, for a week.

Cleanliness is persevered in as much as possible; no urine or stools, but a horrid stinking matter exudes from the penis. At two A. M. the 3d of February, the desire, as well as power to cough, ceased, and shortly after the vomiting; he must, at least, have thrown out, or rather bubbled, and occasionally poured out, to the amount of two quarts. As the cough, &c. ceased, to our great surprize, a great heat, full that of ninety of Fahrenheit took place over the whole body, except very sparingly on the part, and the pulse rose to at least the standard of health in a weakly person; but quick, yet not so quick as when last felt, previous to this, and now about 100. This heat, &c. lasted till four, giving a scalding sensation, and very little moisture; it then relaxed to about eighty, and skin became soft; at six, it was at seventy, and skin greasy, very moist, soft, and putrid like; as even also a blister, which was applied to the sternum, at the early part of the oppressed breath, and short cough; kept open ever since, and did arise not badly, though very slowly; it is now very pale, livid at the edges, and very fetid; the whole a deplorable and extraordinary case.

Had there been the least return of strength, or power of body or mind, the least favourable change in the appearance of the wound, from this accession of heat, unusual and extraordinary as it might first seem to be, I should have been still disposed to hope, catching at any the least gleam of crisis, or force of nature; but it was next to impossible; therefore, we reasonably concluded it to be the last effort of the circulation, or perhaps the last necessary act of it, independent of any attempt to relieve it; to be considered as a simple effort of the laws of the animal economy; the circulation being now principally obstructed in the large vessels of the internal circulation of the viscera, the heart was surcharged, was excited to its last strength, and threw an unusual proportion of blood into the extremities and surface; and the veins getting filled without power to empty themselves, and the heart less able every moment to exert itself, though more required so to do; of course a great quantity of blood was detained on the surface, and communicated the heat spoken of. From six it gradually abated, and pulse became imperceptible, and the whole appearances reverted to the previous state of prostration, and sinking.

There appeared, in the first instance, a little more susceptibility to pain on pressure of the abdomen, and to heat of the poultices; but as soft and loose as before. At nine he began to take long gasps, and at ten breathed his last, after a little contortion of the face and hands; and after a highly distressing picture of the struggles of a stout man, in the vigour of life, against as fatal, as it was sudden, unexpected, and to me still, inexplicable case.

He had been rather addicted to dram-drinking; and by being at sea many years, probably had a degree of scorbutic diathesis; but I never knew him ill except of a cold; always always florid, stout, and strong; no carbuncles, or œdema; nor pains, or discolourations; usually indicative of a diseased liver, or of other viscera, nor of scurvy; his breath always fetid, but I think rather from bad teeth; on the whole, a man, even with the previous state of the affection of the urinary organs, on whose life I would have insured in preference to most.

I shall proceed now to try the question between all the external marks I have related, and the reasoning, observations, axioms, and rules of diagnosis on which we are taught to act; but which I fear in too many instances are blind guides. I may be told this is a single case; but it is such a one, so totally adverse to our presumed knowledge, that while I beg the favour of the medical public at large to criticise on it, and hope to leave through your publication a rational, if not a demonstrative evidence of the non-agreement between cause and effect, taken either from the principles of pathology now admitted, or from others, which may be in the understandings of the learned. I at the same time beg to advance, that experience alone is the school, the test, and only solid foundation of what we should take upon ourselves to say *we know*.

I fear I shall become a greater sceptic than ever in dogma; and that painful as the thought is, and costly to poor human nature; yet that we can be useful to others, only by the collected resolutions, hereafter formed by minute observations in our individual practice; that systems are to be followed with caution by all, and not in the least by those who have not bought their knowledge from practice, consequent on collective, acquired, information of their art, which may be demonstrated; except in the most simple self-evident affections, where indeed nature needs us not, and we had better stand by only to observe; removing what is in her way, and giving warning of danger. I think this case to be on the whole so striking, that it strikes at the very fabric of the first part of theoretic medicine; namely, inflammation. The introductory guide, and companion of most diseases, and of the principles of practice we thence deduce.

Inflammation in all its points of view, of cause and effect, is, I fear, now only the pretended illustration of the infirmities, which come under the care of the physician. It is to be earnestly sought after, but much to be feared, that it ever will be so learnt, to be in medicine, what anatomy is in surgery. I may be told by some, that theories are formed from the accumulated evidences of past experience; some of them may; but parts even of those may be deficient' let alone others which are mere phantoms of the brain and deductive reasoning, as it is called; but the very best are formed from the results of men of different ages, different capacities, and opinions; besides the errors of representation, which obscure them. A system should be the work of one man, from his actual sight, and not allowed to be established but by dissection in all organic affections, and in more general affections as of fever, by almost invariable success! The constant change and variety of theories, and modes of obtaining the same end, especially in general disease, leave it the opprobrium of medicine to have quackery for its essence; that nature, I mean unobstructed nature, and without drugs, might do as well, and, perhaps, did better in the days of Hippocrates and Sydenham, than in those days of boasted perfection.

To put aside a great part of the objection of one man's inability to form an entire system; I answer, let the profession be more divided, let him direct his inquiries to one, or a few particular points. He who shall establish a clear, applicable system of inflammation, will deserve the first laurel, and will pave the way for another to be crowned for his elucidation of fever. Can the same then be equally clear as an Occulist, Lithotomist, Dentist, &c. If I am to lose a limb, let me have a good Anatomist, who has performed no operation but amputation. But some men have such vast capacities, that they are equal to all intuitively, and will even audaciously promise a positive cure to the silly and credulous.

I believe it will be allowed, at least so I have learnt, as well as read, that to confess ones ignorance, is the first step to truth, and to follow that confession up with curiosity and ingenuousness, is the surest way both to know ourselves, and others; without which we can never be truly useful to them; nor should we rest satisfied in our own minds, who are so particularly bound to the duties of humanity.*

Appearances

---

* The state of the patient up to 8 P. M. of Feb. 2, presented nothing new, or unexpected, except the survival of him; but with, if it be possible to conceive it, a diminution of strength; this, however, is certain, that we scarce can see a patient weak, but he may become weaker; and on this principle the medicines were continued; the clammy coldness has increased, the face has sunk, there has been little twitchings of the muscles, and some

Appearances on dissection, half an hour after decease, as quick as possible; because if the affected fœtor, and necessity of burial, and also more satisfactory for a true knowledge of the state of the parts, as near to the time of death as possible, so as to get close to the cause, and that no appearance might be imputed to changes taking place after death, often leading to fallacious conclusions: this is particularly the case in respect to marks of the mortification. I observed to my assistants, that already the effects of gangrene were penetrating, or affecting the integuments, for there was a small space, a little blue and yellowish of the abdominal integuments, near the left ilium, and upper part of the thigh. I expected great fœtor so soon as the body

---

some muttering, but no distinct delirium; and only occasional slight hiccup, chiefly after taking the strong medicines. The cough has become much more troublesome, with heavy lifting and panting expirations from the chest, but with increased expectoration, and occasional vomiting, not only of medicine, but of mucus; blackish, thin, fluid, and some sago which he ate yesterday. No urine or stool, but some fetid matter, and even a few drops of urine from the penis, not the least food. Eye almost dead, tongue moist, but black, and is scarce sensible, and seldom looking about; no fœtor of notice. Pulse occasionally felt small indeed, a few beats after coughing, which is almost incessant, and highly distressing; no distension or pain of abdomen on pressure; all lax, flat, and soft; wonderful he has held so long! — Wound has not increased in size, or putrescence, and part of it is a little red, but the edges very bad; the lower edge has the confirmed black, dry sphacelus, no further extent of vesications, or erysipelatous redness. Continued every two hours, warm fermented cataplasms, and lint wet with brandy.

At 4 A. M. very large blisters to the chest on account of the cough and oppression, with the peculiar distressing breathing and expirations usual in putrid fever, but at 8 P. M. not the least sign of its vesication; fully confirming the great loss of excitability. Repeat as before as nearly as possible, but he has almost lost the power of deglutition, and the low delirium is far advanced. A truly melancholy and vexing case. Last night I thought by pressing a little heavier on the abdomen, and region of the bladder, he resisted, and seemed to cry out; a little of susceptibility of the parts supposed to be affected. But where is the urine? As I could do no harm, passed a catheter again on the ground of this little indication of pain; it passed easily indeed to what it had done, almost as it would do in a dead body; not the least evacuation followed, and to the touch all felt as it did on the night of fixing the catheter; it went as far as I could apparently distinguish into the flattened sides of an empty bladder. I occupied the degree of force then supposed to be applied, which to myself and assistants appeared to be quite moderate; also directed it to right groin, and passed it up by elevation of the hand gently, and yet so as not to have any idea of going beyond the bladder. Also passed the finger in the anus; catheter was felt at a little distance, exactly as it may be in an empty bladder; felt the prostrate gland not large, as well as I could judge; and in short all as before, when last examined: left in the catheter untied, without the stillette, may take its chance, as it can do no harm. Wet his lips, and expect death.

body should be opened, and said we shall find, I doubt not, a great part of the bowels in a state of gangrene, the bladder more so, and the stomach also partaking. All the integuments and muscles presented nearly the usual firmness and colour of health when divided. Adeps was firm, and of its natural colour; peritonæum was slightly inflamed, except the part which doubles over the bladder, all which not only united to it, but that most firmly, with very great thickening and layers of adhesions, by great increase of vascularity; very fluid, and minutely ramified, with innumerable vessels; the usual effect of adhesive inflammation, of longer existence than the date of the few days of his illness, I mean since the rigor.

So firmly and extensively was the bladder united to the peritonæum, the rectum, and surrounding membranes, that the bladder and adjacent parts, though the former had scarce a cavity to hold three ounces, nearly filled the pelvis; whose membranes were also highly inflamed. It was impossible to separate the bladder from the adhesions by the hand, and not readily by dissection, yet there was no hardness unusual to excessive inflammation of the kind, not the least approach to schirrosity in the substance, or coverings of the bladder; the whole of those parts exhibiting an actually existing highest degree of inflammation they could bear, without disorganization, and yet that disorganization had begun. Judging from these parts, he seemed to die at the very acme of increased action of vascularity; or, if you please, at the instant of the change of vessels breaking down, and yet that change not fully made, so as to say he died of gangrene of those parts; yet I shall shew by and by, that an incipient state of solution did exist, but so partial, that I can be brought with difficulty to believe that he died of mortification; and on the other hand, the state of the parts give on the whole death from inflammation. Where were the marks on the skin, pulse, strength, &c. &c.? It appears I have given every external mark of putrescency, and not one, except the rigor of inflammation.

The wound in the perinæum was decidedly gangrenous on a sudden, and increased. Did all the symptoms of a general putrid state, arise from the condition of the wound? Why, after three days, did not the bladder and other viscera, partake of the apparent general atony produced in the system? If the wound was too small and insufficient to lead the viscera into the same state; why was not the wound led to partake of the same action of excitement citement with the viscera? and why did not they in that high positive state of inflammation which they were found in, and which must have existed several days, (and judging by its effects of adhesion, &c. much longer than last week) influence the state of the wound, and the general circulation to the same state of excitement; to heat, pyrexia, fullness, and all the usually excepted symptoms of inflammatory fever? Two such opposite states of the system will not be allowed; but we may suppose this high internal inflammation, and chiefly of the bladder first to exist, and that the wound being least able to bear that active rise, rushed first into gangrene; but it did that instantly, and surely it was capable of first bearing a degree of increased action; but no, not the least ever took place; neither redness, fulness, heat, or pain. The poultices being applied every four hours, this would have been seen; and after all, let what date you please be given to the abdominal inflammation. Why? is the grand question, was not this made known by the common and very distinct marks, above all, perhaps, in abdominal inflammation? I speak now of what I have usually seen, always read, and been taught; viz. heat, quick, hard pulse, excessive pain, and in short, by pyrexia in general of the most violent kind: either way it is inexplicable to me. A man dies in a positive and most pointed state of asthenia to all appearances, and proved by the state of the wound; the indications of course were plain. Durst I bleed or evacuate in any way? Could I doubt administering cordials, tonics, and stimulants? And after this, I prove that the state of death of some of the most essential parts of the system, is existing inflammation. On the other hand, if I had found, as I expected, the bladder ulcerated, or broken down in substance, soft, livid, and in short, the usually extensive marks of gangrene in those who die of mortification of the abdominal viscera; how then explain mortification without previous inflammation? It is not like the gangrene of old people, or of the extremities, or solution of continuity from external violence. How then should I have said the circulation was destroyed, the vessels broken down, unless as some ages back referred to, and what I thought highly probable, from the effect of typhus gravior, or malignant fever, where the whole system gives way rapidly, to a cause not yet satisfactorily explained? If it be said after all, that he died of mortification, why was not the death of the wound, and parts adjacent, and that of the abdominal viscera, the bladder in particular, coeval? But I must go go on, to shew that if two actions cannot exist at once, I mean two opposite states of the system, sthenia and asthenia, that is, there is death, and proceeding gangrene of a part on one hand, and general inflammation and fever on the other; and such opposite actions, in parts so near each other as the wound and bladder, and the others not distant either by sympathy or circulation; yet, to shew that tho' the disease of a part did apparently overcome the production of the usual phlegmona to sight and feel, before death; that actually two opposite states did, or had existed: whilst, internally, inflammation was going on most rapidly, one should rather think that the excitement of these great parts should rather command the local disease, and thus the whole system as usually admitted, partake of the diagnosis reasonably to be expected, and by which alone we can act.

The whole of the intestines were very fluid, indeed all their vessels distended, but especially the arch of the colon, which was also very full of air, and some fæces, as also a considerable space of the illium, but the end of the rectum from about six inches, was still more so, and surrounded with layers of adhesions, except in the part forming the anus; about an inch of which was thin, soft, and almost livid. In those highly inflamed parts of the colon and illium, there were numerous spots of purple, fast approaching to gangrene; and I observed that by the time we had done examining, (about an hour after death,) the whole highly red part of the illium had become almost an uniform space of purplish colour. I consider it as certain, that had the patient lived another day, positive and extensive mortification would have been exhibited; but I am now equally persuaded that the general appearances, together with the heat and smell (not in the least putrid) of the viscera, were those of high inflammation, and that the degree of mortification in those spots, were not such as could excite death by mortification; besides, we have had all the signs of prostration ever since twelve o'clock at night of the rigor.

The stomach was immensely distended with air, and about a pint of that blackish, half fæcal, thin, putrid fluid, of which he had vomited, and part of the sago he ate previous to the day of the rigor, but no distinct marks of inflammation. The liver, pancreas, and spleen, natural, the kidneys also; I mean in a general view of the whole; they were very large, ureters very pervious, but rather inflamed, and thickened; no urine in them, but a white purulent matter matter in small quantity; completely pervious into the bladder, which was also quite empty, and clean; but it is likely that part of the matter these last few days from the penis might be from the ureters, rendered fetid, and darker by being mixed with the matter of the abscess. It would not be easy to convey an adequate idea of the state of the bladder, without a drawing; which as I am not able to do, (although one did accompany the first copy of the case,) I shall endeavour to excite in words alone.—The following out-line may assist.

State of the bladder, a longitudinal two-third section, parallel to its flattened sides.

*a*. The bladder.

B. The ureters.

C. The prostate.

D. Piece of prostate, cut off to shew the mouth of the seminal duct into that gland.

E. A large gangrenous spot of dark purple, but not soft or fetid.

F. Corresponding section of the prostate.

G. Marks of ulceration very thin, and blackish by gangrene, which corresponded to the external wound, and through which the urine had passed; being the seat of the old obstruction and ulceration.

*h*. The extent of the apparently forced opening, its mouth extending to the neck of the bladder, near the ulcerated parts, where the catheter is suspected to have passed.

*i*. Mark of the line of thinness in the corresponding one-third section, where the bladder was rather soft, and nearly broken through, half dissolved as if by ulceration; yet yet the coats were not corroded, the thinness seemed to proceed from the outside; this is on the anterior, and rather superior side.

REMARKS.

The large gangrenous spot had a corresponding discoloured spot on the external surface, but the villous coat was not the least broken by it, nor the part soft, or thinner than elsewhere. The whole villous coat was very thick, of a proper colour, and very smooth, except in the places of the rugæ; indeed its only unnatural state was that of thickness, about the 20th of an inch, and much more so at the neck, where its rugæ assist to act on the sphincter. The long line of the supposed opening by the catheter was immediately beneath the villous coat, and being laid open, exhibited the muscular coat, and cellular substance much disorganized, as if bruised; of a blackish-red, but not like that of mortification; exactly like a part violently torn.

It appeared on close examination, that at its mouth, near the ulcerated thin places of the urethra, there had been a large ulcer in particular, on the very spot in which the point of the catheter entering it, readily passed along, and the whole must have been expected by disease of ulceration, yielding as the catheter passed so readily; this, if admitted as possible, without giving pain, or the least anxiety, though retained two hours, explains why no urine entered the catheter when he made it along its sides. Not only the lining membrane of the urethra where the ulcerated spots are shewn, and beyond, towards the penis, full an inch more was destroyed; but in the membranous part of this canal, the whole substance was nearly corroded, especially of course at the very small point, where the urine passed through; but from the bulb of the urethra to full half way up the membranous part, the canal, was thickened almost to schirrosity, large, &c. in proportion to the state of the bladder, and most firmly united to the muscles, and all surrounding cellular substance; so that it was difficult to separate it, or to distinguish one part from another. The whole bladder was at least four times its natural size when empty, and there was not one drop of fluid in it; in short, its size, and connexions with the surrounding inflamed appendages and surfaces, almost filled the cavity of the pelvis. The thickening was principally of the muscular coat, and I am within bounds to say three-quarters of an inch throughout, was the thickness of the vessels, except at the part denoted by the line, where it was about the usual thickness; it felt softish and looked looked very vascular, ready to break through from without inwards, but had no absolute gangrenous appearance. The neck was even thicker than the fundus, and absolutely cut like a piece of thick white tripe. The prostate gland still more resembled that substance in colour and solidity, and was full the size of a common egg.

I here finish a statement of facts; and I hope I have been able to convey all the ideas and observations which occurred to me during the patient's illness, at his death, and not one suggestion by retrospect of the whole. In so writing cases, and putting the whole down at once, we too often, I fear, turn to favourite notions, or force ourselves to appear consistent and wise; after nature, and the disease have said all they can to remove our difficulties, and give us, as we think, a distinct and clear apprehension of the whole. I confess my apprehension is not so bright; but I endeavoured to dispel its obscurity by minute daily observation, I have therefore preferred giving a copy of a Journal, as a fair ground for the judgment of others. Such forms of recitement, it is well known, cannot have that correctness of expression, or methodized language necessary to please those who read rather for words than facts. I therefore trust that the critisism I court will be only extended to the latter.

*Plymouth, October* 14, 1807.

## Figures and Tables

**Figure f1:**